# The Relationship between RUNX3 Expression, Nursing Strategies and Nutritional Status in Elderly Patients with Advanced Gastric Cancer

**Published:** 2017-06

**Authors:** Wen SONG, Wenhui TENG, Xinyan SHI, Xiaozhen LIU, Zheng CUI, Zibin TIAN

**Affiliations:** 1. Gastrointestinal Endoscopy Center, the Affiliated Hospital of Qingdao University, Qingdao, Shandong, PR China; 2. Operation Room, the Affiliated Hospital of Qingdao University, Qingdao, Shandong, PR China; 3. Dept. of Business, the Affiliated Hospital of Qingdao University, Qingdao, Shandong, PR China; 4. Dept. of Pediatrics, the Affiliated Hospital of Qingdao University, Qingdao, Shandong, PR China; 5. Dept. of Gastroenterology, the Affiliated Hospital of Qingdao University, Qingdao, Shandong, PR China

**Keywords:** Nutritional status evaluation, Advanced gastric cancer, RUNX3, Holistic nursing

## Abstract

**Background:**

The aim of this study was to explore the relationship between nutritional status and expression of RUNX3 in gastric cancer cells and to investigate the effects of nursing strategies on the nutritional status of elderly patients with advanced gastric cancer.

**Methods:**

Forty-eight elderly patients admitted at Affiliated Hospital of Qingdao University with advanced gastric cancer and 30 healthy controls were selected as subjects from 2014–15. The correlation between RNX3 gene expression and nutritional status of the gastric cancer patients was investigated. The patients with advanced gastric cancer who had low expression of RUNX3 gene were treated with holistic nursing while routine nursing was taken for those patients who had normal or high expression of RUNX3 gene. The nutritional statuses of these patients were evaluated after 3 months of nursing. After a follow-up of 1 year, the influence of different nursing methods on the survival time was evaluated.

**Results:**

Compared with normal gastric tissue, the expression of RUNX3 gene and protein in tissues of advanced gastric cancer were significantly decreased (*P*<0.01). Compared with patients with normal or high expressions of RUNX3, the nutritional statuses of advanced gastric cancer patients with low expressions of RUNX3 were lower (*P*<0.01). The nutritional statuses of patients with low expressions of RUNX3 were notably improved after holistic nursing, becoming equivalent to those with normal or high expression of RUNX3 who received routine nursing (*P*>0.05). The survival time of patients with low expression of RUNX3 who received holistic nursing were similar to patients with normal or high expression of RUNX3 who received routine nursing (*P*>0.05).

**Conclusion:**

RUNX3 is correlated with the occurrence and development of advanced gastric cancer. The low nutritional status of elderly advanced gastric cancer patients with low expressions of RUNX3 can be significantly enhanced by holistic nursing, thereby prolonging survival time.

## Introduction

Gastric cancer is one of the most common malignant tumors of the digestive system. It can occur at any age and poor dietary habits are a predominant risk factor for its development (1, 2). With advances in medical technology, both cancer detection rate and treatment rate have increased significantly. However, given that confirmation of diagnosis of gastric cancer in elderly patients tends to occur at the advanced stage and accompanied by metastasis, the cure rate for chemotherapy as treatment is poor (3, 4). Furthermore, the inadequate food intake of elderly patients caused by poor appetite during the chemotherapy period leads to malnutrition, which seriously affects the quality of life and therapeutic effect (5). With the development of genomics, research into the mechanisms of incidence and development of cancer is gradually expanding, with the identification of a large number of implicated tumor suppressor genes and cancer-inducing genes (6, 7).

There is a close relationship between the expression of the RUNX3 gene and gastric cancer, indicating that RUNX3 can affect the progression of gastric cancer by regulating the proliferation and migration of gastric cancer cells (8). The effect of nursing practices on clinical treatment of the disease has also received increasing attention. A large body of evidence has revealed that incorporating holistic nursing of psychological and physical components into routine ward nursing can effectively improve the quality of life and enhance the survival time for patients (9, 10).

This study aimed to assess the nutritional statuses of elderly patients with advanced gastric cancer, and summarily analyze its relationship with RUNX3 expression level and nursing strategies. This may provide a theoretical and practical basis for the treatment and nursing practices for elderly patients with advanced gastric cancer.

## Methods

### Patients

Study samples were all selected from elderly patients with advanced gastric cancer admitted to the Affiliated Hospital of Qingdao University for treatment, from Feb 2014 to Feb 2015. All patients had a diagnosis of advanced gastric cancer confirmed by gastroscopy and pathological examination. The above cases were screened and 48 patients who conformed to the criteria were selected. Among them, there were 25 males and 23 females, with an age range of 63 to 87 years. Patients with comorbid conditions of the heart, lung, liver, renal and other systems were excluded. Tissue that was more than 3 cm away from the surgical margins of gastric cancer site was considered adjacent tissue of cancer; no cancer cells were found by pathological examination of the adjacent tissue. Meanwhile, 30 normal elderly patients who had gastric tissues without cancer cells as examined by pathology during the same period were selected as the control group. All selected patients were followed up in our hospital for one year and treatment regimens were strictly adhered to over this duration.

### Detection of RUNX3 expression level

After gastric cancer tissues and adjacent tissues of the group with advanced gastric cancer and normal gastric tissue of the control group were obtained, total RNA was extracted by TRIzol Kit (Invitrogen, Carlsbad, CA, USA) with strict accordance to the operating instructions. Using agarose gel electrophoresis, the bands of 28S and 18S were clear, and 28S had twice the brightness of 18S. After the integrity of RNA was confirmed, reverse transcription was conducted using reverse transcription kit (Invitrogen) with strict accordance to protocol to obtain cDNA. The expression of RUNX3 in each tissue sample was detected by semi-quantitative PCR with β-actin as internal reference. The reaction conditions were as follows: 95 °C for 5 min, 95 °C for 1 min, 65 °C for 35 s and 72 °C for 30 s, for 30 cycles. The primer sequences were shown in Table 1 and the primers were synthesized by Tiangen (Beijing, China). The agarose gel electrophoresis was used after the reaction and the products were then observed by an ultraviolet imaging system. Lysate was added into the above samples of gastric cancer tissues, adjacent tissues of cancer and normal gastric tissues according to the proportion of 1:20, followed by the addition of homogenate. After centrifugation, the whole supernatant was transferred out and total protein was obtained. Through protein quantification, the protein samples were prepared into the loading systems with equal concentrations. In accordance with western blot methods, after electrophoresis, transferal to the membrane and sealing off were performed in the proper sequence for the samples. Then, the specific monoclonal antibody of RUNX3 (Cell Signaling Technology, Danvers, MA, USA) was incubated at 4 °C overnight and a secondary antibody (Cell Signaling Technology) was then incubated at room temperature for 2 h, followed by development and exposure in a dark room. The corresponding protein bands were obtained, which were analyzed after scanning, with β-actin as an internal reference. RUNX3/β-actin was selected as an indicator to detect the relative expression levels of RUNX3 protein in the gastric tissues.

**Table 1:** PCR primers used in this study

	**Sequence**
RUNX3	Forward primer: 5′-TTATGAGGGGTGGTTGTATGTGGG-3′
	Reverse primer: 5′-AAACAACCAACACAAACACCTCC-3′
β-actin	Forward primer: 5′- GATGATTGGCATGGCTTT-3′
	Reverse primer: 5′- CACCTTCCGTTCCAGTTT-3′

### Intervention of holistic nursing strategies Psychological nursing

For patients with cancer, resistance to treatment caused by the unknown and anxious psychology and emotions can lead to their refusal of chemotherapy. Therefore, it is necessary to communicate deeply with patients, listening to the patients’ complaints and concerns carefully and answering their questions. This enables them to understand the therapeutic effects and reduce the patients’ concerns and psychological conflicts as much as possible, which can enhance their confidence in overcoming the disease and encourage them to cooperate with treatment.

### Intestinal tract and diet nursing

Adverse reactions caused by chemotherapeutics often leads to considerable gastrointestinal discomfort in patients receiving chemotherapy, which can affect food and nutrient intake. Thus, gastrointestinal adverse reactions were closely monitored during chemotherapy. This entailed ensuring that the intake of hard or irritating foods was avoided and nutritious foods, as well as sufficient water, were made readily available for consumption. For patients with intolerance, appropriate drugs were administered for symptomatic treatment, to increase patients’ compliance of food intake.

### Deep vein indwelling catheter nursing

Strict aseptic clinical practices were adopted in order to prevent infection at the postoperative puncture indwelling site of patients and the patency of catheter was maintained to avoid blockage. If leakage of chemotherapy drugs at the indwelling catheter site occurred, a local cold compress was utilized to reduce leakage, followed by hydropathic compress after 24 h to promote the absorption of seepage.

### Nutritional status evaluation SGA scoring method

The self-rating scale, Subjective Global Assessment (SGA), was used to evaluate the nutritional status levels of patients. After statistical analysis of the scores, the nutritional status levels were divided into three grades: SGA-A (normal nutrition), SGA-B (mild to moderate malnutrition) and SGA-C (severe malnutrition) (11).

### Anthropometry

Triceps skinfold thickness (TSF) was the main measurement index of anthropometry. During measurement, the surveyor stood behind the patient and ensured the patient hung down the upper arms naturally. The skin at the midpoint of the backside of the left arm together with subcutaneous tissue of patient was pinched up by the surveyor, into a fold. Then, the thickness of pinched subcutaneous tissue and fat was measured by a TSF measurement device. For males, TSF value > 10.17 was normal, 6.78 < TSF < 10.17 was regarded as mild - moderate malnutrition and TSF < 6.78 was considered as severe malnutrition. For females, TSF value > 13.41 was normal, 8.94 < TSF < 13.41 was regarded as mild - moderate malnutrition and TSF < 8.94 was considered as severe malnutrition (12).

### Laboratory testing

Serum prealbumin levels of patients were detected using Elisa kit. With prealbumin (PA) and albumin (ALB) as the evaluation indices, standard values of PA > 200 mg/L and ALB > 359 mg/L were used as boundary values in diagnosing degrees of malnutrition.

### Statistical analysis

SPSS 19.0 software (SPSS, Inc., Chicago, IL, USA) was used for statistical analysis and data processing. The data were expressed as mean ± standard deviation. Measurement data was compared using t test and enumerable data between groups was compared using the chi square test. Survival analysis was carried out using the Kaplan-Meier method, followed by the Log-rank test. *P<*0.05 suggested that a difference was statistically significant.

## Results

### RUNX3 expression in tissues of elderly patients with advanced gastric cancer

**Fig. 1:**
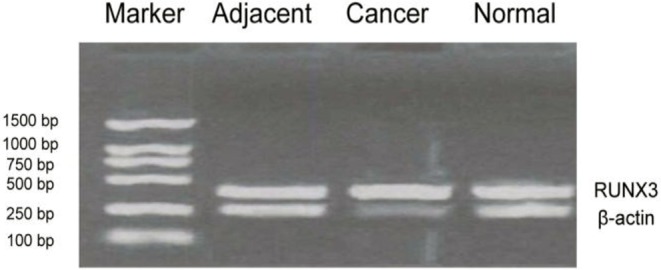
Detection of RUNX3 expression levels using semi-quantitative PCR. Compared with adjacent tissues of cancer and normal gastric tissues, the expression levels of RUNX3 gene in advanced gastric cancer tissues were significantly decreased (*P*<0.01)

**Fig. 2:**
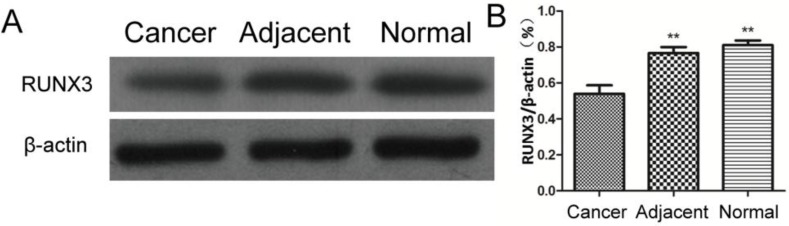
Detection of RUNX3 protein expression by using Western-blot. A: The results of expression detected by western blot. B: The relative expression levels of RUNX3. Compared with adjacent tissues of cancer and normal gastric tissues, the expression levels of RUNX3 protein in advanced gastric cancer tissues were significantly decreased, ^**^P<0.01

Forty-eight samples of cancer tissues and 48 samples of adjacent normal tissues from elderly patients with advanced gastric cancer and 30 samples of normal gastric mucosa tissues from healthy controls were selected. The RUNX3 expression levels of mRNA, as detected by semi-quantitative PCR, were shown in Fig. 1. Compared with tissues adjacent to cancer and normal gastric tissues, RUNX3 gene in tissues of advanced gastric cancer showed lower levels of expression of RUNX3 (*P*<0.01). The expression levels of RUNX3 protein, as detected by western blot, are shown in Fig. 2. Compared with tissues adjacent to cancer and normal gastric tissues, RUNX3 protein in tissues of advanced gastric cancer showed lower levels of expression (*P*<0.01).

### Relationships between RUNX3 expression and the clinical features of patients

The correlation between the expression of RNX3 in advanced gastric cancer tissue and tumor size, as well as other clinical features, of elderly patients with advance gastric cancer was investigated. As shown in Table 2, there were significant correlations between RUNX3 expression and tumor size (*P*=0.0086), clinical staging (*P*=0.0079), lymphatic metastasis (*P*=0.0063) and tumor recurrence (*P*=0.0056).

**Table 2:** Relationship between the relative expression of RUNX3 and clinical features of patients

**Classification**	**n**	**Relative expression of RUNX3**	***P***
Gender			
Male	25	0.467±0.121	0.0863
Female	23	0.496±0.113	
Age			
> 70 years	42	0.485±0.109	0.0792
≤ 70 years	6	0.502±0.117	
Tumor size (cm)			
≤ 5	35	0.787±0.114	0.0086^**^
> 5	13	0.426±0.103	
Clinical staging			
Stage I – II	9	0.815±1.23	0.0079^**^
Stage III – IV	39	0.328±1.17	
Lymphatic metastasis			
Yes	41	0.281±0.122	0.0063^**^
No	7	0.732±0.156	
Recurrence			
Yes	32	0.359±1.52	0.0056^**^
No	16	0.987±1.69	

Note: Expression of RUNX3 was significantly lower among the adverse classification of each clinical indicators, including tumor size, clinical staging, metastasis and recurrence (^**^P<0.05).

### Evaluation of nutritional of patients and the relationship with RUNX3 expression

Patients of the advanced gastric cancer group were divided into a low expression group (n=35) and a normal or high expression group (n=13), according to the expression of RUNX3. The nutritional status of patients was evaluated by the self-rating scale of SGA, TSF measures of anthropometry and serum prealbumin concentrations, the results of which are shown in Table 3. All of the nutritional status indicators were significantly lower among the group with low expression of RUNX3 compared to normal or high expression (*P*<0.05).

**Table 3:** Relationship between nutritional status evaluation and RUNX3 expression in patients with advanced gastric cancer

**Evaluation index**	**RUNX3 low expression group (%)**	**RUNX3 high expression group (%)**	***P***
SGA-A	5.7	38.5	< 0.05^*^
SGA-B	54.3	38.5	
SGA-C	40.0	23.0	
TSF: normal	8.5	46.2	< 0.01^**^
TSF: mild - moderate malnutrition	57.2	38.5	
TSF: severe malnutrition	34.3	15.3	
PA - normal group	5.7	61.5	< 0.01^**^
PA - malnutrition group	94.3	38.5	
ALB - normal group	8.5	69.2	< 0.01^**^
ALB - malnutrition group	91.5	30.8	

Note: The results of the self-rating scale of SGA showed that the nutritional statuses of patients with low expressions of RUNX3 were significantly lower (^*^P<0.05). The results of TSF and serum prealbumin indicated that the nutritional statuses of patients with low expressions of RUNX3 were significantly lower (^**^P<0.01).

### Reevaluations of the nutritional status of patients following implementation of holistic nursing practices

The elderly gastric cancer patients were each treated with the same treatment regimen. However, the patients with low expression of RUNX3 were given holistic nursing while routine nursing practices were applied for patients who had normal or high expressions of RUNX3. The nutritional statuses of the patients were evaluated after 3 months using SGA, TSF and serum prealbumin, and were expressed as the percentage of the cases (low expression vs. normal or high expression) within each category of nutritional status out of total cases, as shown in Table 4. The results of self-rating scale of SGA, TSF and serum prealbumin showed that the nutritional statuses of patients with low expression of RUNX3 were notably improved after holistic nursing, to levels that were equivalent to those of patients with normal or high expressions of RUNX3 and who received routine nursing. None of the differences were statistically significant (*P*>0.05), indicating that holistic nursing practices improved the nutritional status of those with low expression.

**Table 4:** Evaluation of nutritional statuses of patients after holistic nursing

**Evaluation index**	**Holistic nursing group (low expression of RUNX3) (%)**	**Routine nursing group (normal or high expression of RUNX3) (%)**	***P***
SGA-A	43.9	38.5	> 0.05
SGA-B	34.6	38.5	
SGA-C	21.5	23.0	
TSF: normal	42.5	46.2	> 0.05
TSF: mild - moderate malnutrition	37.2	38.5	
TSF: severe malnutrition	20.3	15.3	
PA - normal group	59.7	61.5	> 0.05
PA - malnutrition group	40.3	38.5	
ALB - normal group	63.5	69.2	> 0.05
ALB - malnutrition group	36.5	30.8	

Note: The results of self-rating scale of SGA, TSF and serum prealbumin showed that the nutritional statuses of patients with low expression of RUNX3 after holistic nursing practices were equivalent to those of patients with normal or high expression of RUNX3 and who received routine nursing practices (*P*<0.05)

### Survival analysis

**Fig. 3:**
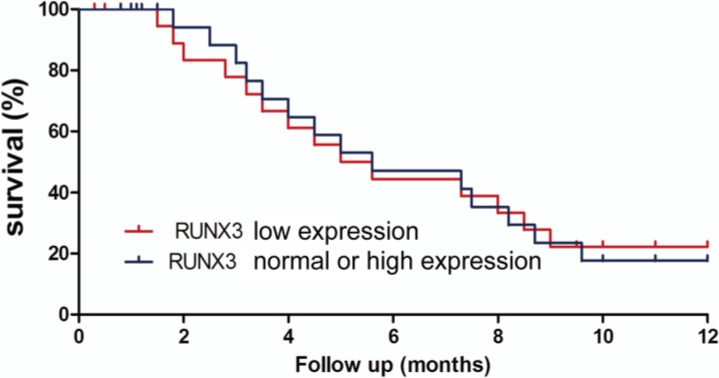
Relationship between RUNX3 expression levels and the survival time of patients. The survival time of patients with low expressions of RUNX3 after receiving holistic nursing were equivalent to those of patients with normal or high expressions of RUNX3 after receiving routine nursing, showing no statistically significant differences (*P*>0.05)

After a follow-up period of one year of the patients with advanced gastric cancer, comprehensive clinical data was collected and the survival time of patients in the two groups were compared. As shown by the results of the survival analysis in Fig. 3, there was no significant difference in survival time between patients with low expression of RUNX3 and patients with normal or high expressions.

## Discussions

With ongoing developments in molecular biology, investigation into the pathogenesis of gastric cancer has gradually revealed details into molecular mechanisms of its etiology. However, there is a lack of definitive evidence associating specific molecules with cancer cell formation. At present, scholars consider gastric cancer to be a complex process involving multiple genes, including the interaction between proto-oncogenes and tumor suppressor genes (13, 14). Researchers generally believe that the occurrence of various tumors is closely related to the reduction in the activity or inactivation of tumor suppressor genes; moreover, the inactivation of tumor suppressor genes often leads to tumor progression and metastasis (15). Research on the correlation between RUNX3 and the occurrence of gastric cancer has garnered increasing scientific attention. The expression of RUNX3 is abnormal in most gastric cancer cells and mediates the progression of gastric cancer (16). TGF-β had a definite role in inhibiting the occurrence of tumors; moreover, as a transcription factor in a downstream part of the TGF-β signaling pathway, the reduction of RUNX3 expression could lead to a disruption in the downstream signaling pathway and induce the occurrence of multiple tumors (17).

The expression of RUNX3 gene and RUNX3 protein in elderly patients with advanced gastric cancer were detected by semi-quantitative PCR and western blot, respectively, in this study. The results demonstrated that, compared with tissues adjacent to cancer and normal gastric mucosa tissues, the RUNX3 gene in tissues of gastric cancer had lower levels of expression (*P*<0.01). This suggests that the expression of RUNX3 is correlated with the occurrence of gastric cancer, such that a reduced expression of RUNX3 may facilitate the development of gastric cancer. It also indicates that RUNX3 may function as a tumor suppressor gene for gastric cancer. The results of western blot showed that, compared with tissues adjacent to cancer and normal gastric mucosa tissues, RUNX3 protein in tissues of gastric cancer had lower levels of expression (*P*<0.01).

The interaction of SMAD and RUNX protein activated target genes, which could regulate the apoptosis and degeneration of cells. Moreover, the SMAD compound relied on the involvement of RUNX3 protein for its activity (18). Thus, current research supports the theory that the expression of RUNX3 protein is closely correlated with the occurrence of gastric cancer.

The nutritional statuses of patients were comprehensively evaluated by the self-rating scale of SGA, TSF and serum prealbumin in this study. The results indicated that the nutritional statuses of patients in the group with low expression of RUNX3 were significantly lower than patients with normal or high expression (*P*<0.01). This suggests that malnutrition is more prevalent in gastric cancer patients and the severity is closely related to the expression of RUNX3. As a method to evaluate nutritional status, SGA is characterized as a simple, rapid and non-invasive tool for malnutrition assessment. Anthropometry estimation by TSF is an important method to evaluate the nutritional statuses of patients, with the advantages of reasonably high accuracy and strong feasibility. Whether there is negative, nitrogen balance in patients could be assessed with high sensitivity by serum prealbumin detection. Protein decomposition is greater than synthesis when the body is in a stress stage, so plasma prealbumin will change before the reduction of plasma albumin. In this study, by adopting the above three different methods to comprehensively evaluate nutritional status, the overall nutritional statuses of patients could be effectively obtained with precision and accuracy.

Holistic nursing, as applied in this study, included special nursing practices directed towards the psychological and nutritional needs of each patient, as well as focused attention on intestinal symptomology and indwelling venous catheter maintenance, in addition to routine ward nursing. For patients with low expression of RUNX3, holistic nursing can improve the patients’ initiative and acceptance of treatment, mentally and physiologically, thereby enhancing patients’ compliance for treatment. The nutritional statuses of patients receiving holistic nursing in the group with low expression of RUNX3 and patients receiving routine nursing in the normal or high expression group were comparable after 3 months of follow-up (*P>*0.05), with the low expression group showing a notable increase in nutritional status. The results indicate that nursing can effectively reduce patients’ anxiety and depression related to their diagnosis and improve their acceptance and compliance to treatment, thereby enhancing their nutritional status and quality of life and prolonging their survival time. A large body of evidence from China and other countries also supports that the application of comprehensive holistic nursing practices on disparate diseases can significantly reduce patients anxiety, establish their confidence for treatment and improve both their quality of life and family cohesion (19, 20).

## Conclusion

The expression of RUNX3 is closely correlated with the occurrence and development of advanced gastric cancer in elderly patients. Nutritional status is lower among patients with low expression of RUNX3, an indicator that can be significantly enhanced by holistic nursing, thereby prolonging survival time. These results provide a theoretical basis and practical significance for holistic nursing care for elderly patients with advanced gastric cancer in clinical settings.

## Ethical considerations

Ethical issues (Including plagiarism, informed consent, misconduct, data fabrication and/or falsification, double publication and/or submission, redundancy, etc.) have been completely observed by the authors.
